# The Vasodilatory Effects of Anti-Inflammatory Herb Medications: A Comparison Study of Four Botanical Extracts

**DOI:** 10.1155/2017/1021284

**Published:** 2017-11-28

**Authors:** Hong Ping Zhang, Dan-Dan Zhang, Yan Ke, Ka Bian

**Affiliations:** ^1^National Clinical Research Base of Traditional Chinese Medicine, The Affiliated Hospital of Traditional Chinese Medicine of Xinjiang Medical University, Urumqi 830011, China; ^2^Murad Research Institute for Modernized Chinese Medicine, Shanghai University of Traditional Chinese Medicine, Shanghai 201203, China; ^3^Teaching Experimental Center, Shanghai University of Traditional Chinese Medicine, Shanghai 201203, China; ^4^Department of Biochemistry and Molecular Medicine, George Washington University, Washington, DC 20052, USA; ^5^George Washington Cancer Center, Washington, DC 20052, USA

## Abstract

Inflammation plays a pivotal role in the development and progression of cardiovascular diseases, in which, the endothelium dysfunction has been a key element. The current study was designed to explore the vasodilatory effect of anti-inflammatory herbs which have been traditionally used in different clinical applications. The total saponins from* Actinidia arguta* radix (SAA), total flavonoids from* Glycyrrhizae* radix et rhizoma (FGR), total coumarins from* Peucedani* radix (CPR), and total flavonoids from* Spatholobi* caulis (FSC) were extracted. The isometric measurement of vasoactivity was used to observe the effects of herbal elements on the isolated aortic rings with or without endothelium. To understand endothelium-independent vasodilation, the effects of herb elements on agonists-induced vasocontractility and on the contraction of endothelium-free aortic rings exposed to a Ca^2+^-free medium were examined. Furthermore, the role of nitric oxide signaling in endothelium-dependent vasodilation was also evaluated. In summary, FGR and FSC exhibit potent anti-inflammatory effects compared to CPR and SAA. FGR exerts the strongest vasodilatory effect, while CPR shows the least. The relaxation induced by SAA and FSC required intact endothelia. The mechanism of this vasodilation might involve eNOS. CPR-mediated vasorelaxation appears to involve interference with intracellular calcium homeostasis, blocking Ca^2+^ influx or releasing intracellular Ca^2+^.

## 1. Introduction

Inflammation plays a pivotal role in the development and progression of several cardiovascular diseases, including atherosclerosis [1]. Numerous epidemiologic studies support the concept that vascular inflammation correlates with an increased risk of atherosclerosis [2]. While inflammation contributes to cardiovascular pathology, the question remains whether inhibition of inflammation prevents or even reverses the progress of vascular diseases. Multiple clinical studies have shown that the use of statins reduces cardiovascular morbidity and mortality [3, 4]. However, a direct test of the inflammatory hypothesis of cardiovascular disease requires an agent that can inhibit inflammation without affecting other components of atherothrombosis, while also exhibiting an acceptable safety profile. To address this issue, a cardiovascular inflammation reduction trial (CIRT: ClinicalTrials.gov.ID# NCT01594333) has begun at the Brigham and Women's Hospital and the National Heart, Lung, and Blood Institute (NHLBI) that proposes the use of very-low-dose-methotrexate (VLDM, 10 mg weekly) on 7,000 patients with stable coronary artery disease and persistent elevations of high-sensitivity C-reactive protein (Hs-CRP). Despite its anti-inflammatory effects, methotrexate is an antimetabolite drug that is used to treat cancers and has significant side effects at high doses. Therefore, alternative therapeutic options should be considered.

The anti-inflammatory traditional Chinese medicines as well as botanic elements have been studied for years [5–8]. On the other hand, botanically derived elements have been recognized for the beneficial effect on cardiovascular and metabolism systems [9, 10]. To further explore the potential of using those agents on the cardiovascular system, we studied the vasodilatory effects of four of these herb elements and reviewed their medical applications (Table 1).* Actinidia arguta* radix (Tengligen) is a member of the Actinidiaceae family. Pharmacology research has revealed anticancer, immune regulation and hypotensive activity from* Actinidia arguta* radix [11, 12]. To the best of our knowledge, there are no scientific reports on the blood-pressure lowering mechanisms of any* Actinidia arguta* radix extracts.* Glycyrrhizae *radix et rhizoma (Gancao) has multiple therapeutic uses, some of which are related to its anti-inflammatory properties. These include treating cough, relieving pain, clearing heat, and eliminating toxins and poisons [13]. Modern pharmacology research has also reported that the flavonoids from* Glycyrrhizae *radix et rhizome (FGR) have antioxidant properties [14] with therapeutic benefits including the inhibition of cough and treatment of bacterial infections [15, 16]; however, its effects on vascular contractility is unknown.* Peucedani* radix (Qianhu) has been an important agent for treating respiratory symptoms and diseases through the centuries. This herb is traditionally characterized as dispelling wind and removing heat, relieving cough, and resolving phlegm [17] and has been used to relieve the symptoms of influenza and asthma.* Peucedani* radix has strong anti-inflammatory properties as one of its therapeutic mechanisms [18, 19]. Many coumarin constituents have been extracted from this herb and they are reported to be responsible, in major, for its biological activity [20]. It has been noted that* Peucedani* radix can exert beneficial effects in hypoxic pulmonary hypertension [21]. However, the action of this drug on the circulatory system is largely unknown.* Spatholobi* caulis (Jixueteng) has been traditionally used for irregular menstruation, numbness, and inflammatory arthralgia [22]. According to pharmacology research, it can reduce oxidative stress [23] and inflammation [24]. Several studies have demonstrated in vitro and in vivo cytotoxic effects of* Spatholobi *caulis extracts on tumor cells [25–28]. It has been suggested that the flavonoids of* Spatholobi* caulis (FSC) are the major active components of its therapeutic actions [29]. A few studies have demonstrated the impact of* Spatholobi* caulis on cardiovascular conditions. This botanical agent interferes with platelet aggregation via interference at the glycoprotein IIb/IIIa receptor [30]. It has also been shown to reduce plasma lipid levels in hyperlipidemic quail [31]. In a rat model of cerebral ischemia, Lee et al. found a significant increase in cerebral blood flow after treatment with* Spatholobi* caulis [32, 33]. Although it is speculated that the blockage of calcium channels is responsible for this action [34], a further vascular pharmacological study of this botanical agent is warranted.

In the present study, we examined anti-inflammatory effects of the four botanical extracts by using LPS and IFN-*γ*-stimulated macrophages. The isometric vasoactivity was measured to evaluate the vasodilating properties of the extracts on isolated rat thoracic aortas. The action mechanisms were explored through pharmacological examinations.

## 2. Methods and Materials

### 2.1. Herbs and Chemicals

Herbs were purchased from Shanghai Yang He Tang TCM Pieces, Ltd. Company (Shanghai, China) and authenticated by the Shanghai Institute of Food and Drug Control. Acetylcholine (Ach), phenylephrine (PE), NG-nitro-L-arginine methyl ester (L-NAME), indomethacin (Indo), 1H-[1,2,4]-oxadiazole-[4,3-a]-quinoxalin-1-one (ODQ), glibenclamide (Glib), tetraethylammonium (TEA), prostaglandin 2*α* (PG_2*α*_), BaCl_2_, angiotensin II (AngII), 5-hydroxytryptamine (5-HT), dopamine (Dopa), endothelin-1 (ET-1), RPMI 1640 medium, IFN-*γ*, and lipopolysaccharide (LPS) were all purchased from Sigma Chemical Co. (St. Louis, MO, USA). Ethylene glycol bis (2-aminoethyl ether) tetraacetic acid (EGTA) and other inorganic salts were all purchased from Sinopharm Chemical Reagent Co., Ltd. (Batch number F20060620). Ach, PE, TEA, AngII, 5-HT, dopamine, and ET-1 solutions were prepared with distilled water. Glibenclamide and ODQ solutions were prepared with DMSO. Control experiments demonstrated that the highest DMSO concentration (1 : 400) had no effect on vascular tone.

### 2.2. Cell Cultures

RAW 264.7 cells were used in current study for the following considerations. First, the RAW 264.7 cell line is a pure clone that can be grown in a pretty much identical and indefinitely manner which is necessary for our drug screen platform. Second, RAW 264.7 cells are transformed and are not functional for certain signaling pathways such as activated inflammasomes [35], which will benefit the purpose of our designed study in which anti-inflammatory effect of the herbs will be evaluated. RAW 264.7 cells were obtained from the American Tissue Culture Collection. The cells were maintained in complete RPMI 1640 media supplemented with 10% heat-inactivated FBS and 1.5% sodium bicarbonate at 37°C in a humidified 5% CO_2_ atmosphere. Cells were plated at a density of 1 × 10^5^ cells/well in 96-well plates or 2 × 10^6^ cells in each 30 mm dish and allowed to attach for 2 hours. For stimulation, the media were replaced with fresh RPMI 1640, and the cells were then stimulated with 10 U/ml of IFN-*γ* and 100 ng/mL of LPS in the presence or absence of FGR for the indicated periods.

### 2.3. Experimental Animal and Blood Vessel Ring Preparations

Male Sprague-Dawley rats (250–300 g) were obtained from Shanghai Slac Experimental Company, Ltd. (Shanghai, China). The animal procedures were carried out in strict accordance with the* Guide for the Care and Use of Laboratory Animals* (Shanghai University of Traditional Chinese Medicine). All experiments were performed under license from the Government of China.

The preparation of the vascular rings was performed as described by Zhang et al. [36]. Briefly, the rats were sacrificed by decapitation and their thoracic aortas were rapidly and carefully dissected away into ice-cold freshly prepared Krebs-Henseleit (K-H) solution. The aortas were cut into ring segments of approximately 3 mm wide. For some aortic rings, the endothelial layer was mechanically removed by gently rubbing the luminal surfaces of the aortic rings back and forth several times.

### 2.4. Recording of Isometric Vascular Tone

Each ring was suspended by means of two L-shape stainless-steel hooks in an organ bath filled with Krebs-Henseleit solution maintained at 37°C while being continuously infused with bubbled 95% O_2_ and 5% CO_2_. The lower hooks were fixed to the bottom of the organ bath and the upper wires were attached to an isometric force transducer connected to a data acquisition system (PowerLab/4P ADInstruments, Australia) for continuous recording of tension. The baseline load placed on the aortic rings was 2.0 g.

Examination of endothelial integrity was performed as described by Xing et al. and others [37–39]. Briefly, endothelial integrity or functional removal was verified by the appropriate relaxation response to 10 *μ*mol/L acetylcholine on 1 *μ*mol/L phenylephrine contracted vessels.

### 2.5. Experimental Protocol

#### 2.5.1. Nitric Oxide Production

RAW 264.7 cells were plated in 96-well plates (1 × 10^5^/well) and stimulated with 100 ng/mL LPS and 10 U/mL IFN-*γ* for 24 h. The cell-free culture media were collected and analyzed for nitrite accumulation as an indicator of NO production using the Griess reagent. The NO assay was performed as described by Zhang et al. [39]. Briefly, 100 *μ*L of Griess reagent (0.1% naphthylethylenediamine and 1% sulfanilamide in 5% H3PO4 solution) was added to an equal volume of supernatant from sample-treated cells. The plates were incubated for 10 minutes and then were read at 540 nm against a standard curve of sodium nitrite. Percent inhibition was expressed as 100 × [1 − (NO release with sample − spontaneous release)/(NO release without sample − spontaneous release)].

#### 2.5.2. Testing the Effects of FGR, FSC, CPR, and SAA on PE-Induced Constriction

The vasodilatory effects of the four botanical extracts were tested in both endothelium-intact and endothelium-denuded rings constricted by PE (1 *μ*mol/L). Once a plateau of PE contraction was attained, each of the botanical extracts was applied cumulatively according to a concentration gradient. At the end of each experiment, forskolin was added to induce blood vessel relaxation and the tension of aortic rings was recorded.

To attempt to understand the mechanisms of vascular relaxation, nitric oxide synthase inhibitor L-NAME, cyclooxygenase inhibitor indomethacin, soluble guanylyl cyclase inhibitor ODQ, adrenergic *β*-receptor inhibitor propranolol, K_ATP_ blocker glibenclamide, K_Ca_ blocker TEA, and K_IR_ blocker BaCl_2_ were individually used to pretreat endothelium-denuded rings for 15 min, respectively, prior to addition of 1 *μ*mol/L of phenylephrine. Afterwards, relaxations induced by each of the botanical extracts were observed, including the concentration-dependent vasodilation.

#### 2.5.3. Measuring the Effects of FGR, FSC, CPR, and SAA on Vasoconstrictors

The endothelium-free aortic rings were first exposed to constrictors at different concentrations. This included Dopa (0.1, 1, 10, 100, and 1,000 nmol/L), 5-HT (10, 100, 1000, 10,000, and 100,000 nmol/L), Ang II (0.1, 1, 10, 100, and 1,000 nmol/L), K^+^ (10.00, 15.85, 25.12, 39.81, 63.10, and 100.00 mmol/L), Vaso (0.1, 1, 10, 100, and 1,000 nmol/L), ET-1 (10, 25, 50, 75, and 100 nmol/L), PG_2*α*_ (1, 10, 100, 1,000, and 10,000 nmol/L), and PE (1, 10, 100, 1,000, and 10,000 nmol/L). After washing, the rings were incubated individually with one of the four botanical extracts at concentrations of EC_50_ for 10 minutes. Contractions induced by vasoconstrictors were again observed. The level of vasoconstriction in response to 60 mmol/L KCl was used as the maximum (100%).

#### 2.5.4. Measuring the Effects of FGR, FSC, CPR, and SAA on Calcium Influx

Endothelium-free aorta rings were washed and treated with calcium-free, high-K^+^ solution (containing 100 *μ*mol/L EGTA and 60 mmol/L KCl). Then, the preparations were incubated and cumulatively exposed to increasing concentrations of CaCl_2_ (0.4, 0.8, 1.2, 1.6, 2.0, and 2.4 mmol/L). The vasoconstrictor responses to CaCl_2_ were compared between four groups using each of the botanical extracts as well as a control group. The level of vasoconstriction in response to 60 mmol/L K^+^ in normal Ca^2+^-media was used as the maximum (100%).

#### 2.5.5. Measuring the Effects of FGR, FSC, CPR, and SAA on Calcium Release

Endothelium-free aortic rings were washed and exposed to calcium-free Krebs-Henseleit solution (containing 100 *μ*mol/L EGTA) for 10 minutes. After this, 1 *μ*mol/L of phenylephrine was added. This resulted in small tonic contractions that were mainly caused by the release of intracellular calcium. Once a plateau of PE contraction was attained, the bath solution was instead in calcium-free Krebs-Henseleit solution (containing 100 *μ*mol/L EGTA) for 5 minutes. Four groups were exposed to each of the botanical extracts at a concentration of EC_50_ in addition to a control group, and these groups were compared. The level of vasoconstriction in response to 60 mmol/L K^+^ in normal Ca^2+^-media is used as the maximum (100%).

#### 2.5.6. Effect of Four Botanical Extracts on Organ Tissue Viability

The effects of four botanical extracts on the viability of freshly isolated aortic organ tissue were tested by repeatedly treating the extracts with the same aortic rings either with or without endothelium. The multiple treatments did not affect the contractility of the vessel induced by 60 mmol/L K^+^. The vasodilation towards acetylcholine of the aortic rings was also intact after several times of applications of botanical extracts.

### 2.6. Statistical Analysis

All of results are expressed as mean ± SD. Statistical significance was analyzed using unpaired Student's *t*-tests for comparisons between two groups. A value of *P* < 0.05 was considered statistically significant.

## 3. Results

### 3.1. FGR, FSC, CPR, and SAA Blocked LPS and IFN-*γ*-Induced NO Production in RAW 264.7 Cells

**Figure 1:**
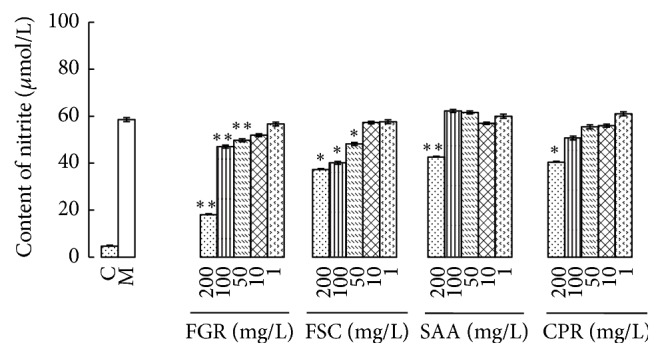
Effects of four botanical extracts on nitrite accumulation in macrophages stimulated by LPS plus IFN-*γ*, ^*∗*^*P* < 0.05, ^*∗∗*^*P* < 0.01 versus model group (M), *n* = 6.

264.7 cells were stimulated by 10 U/ml of IFN-*γ* and 100 ng/ml of LPS that can upregulate the expression of iNOS. iNOS has a key role in inflammatory action. Targeting de novo regulation of iNOS is the therapeutic strategy to cure inflammation-related diseases [40]. RAW 264.7 cells were stimulated by 10 U/ml of IFN-*γ* and 100 ng/ml of LPS with and without pretreatment of four botanical extracts. The concentration of nitrite was measured at 24 hours after the stimulation. As shown in Figure 1, total flavonoids from* Glycyrrhizae *radix et rhizoma (FGR) and total flavonoids from* Spatholobi* caulis (FSC) significantly suppressed the IFN-*γ* and LPS-induced production of NO in a dose-dependent fashion. LPS and IFN-*γ*-induced NO in RAW 264.7 cells were inhibited by FGR and FSC in a concentration-dependent manner. The maximal inhibition achieved (at 200 mg/L) was 75.06% and 39.44%, respectively, for the two drugs. However, higher concentrations of total saponin from* Actinidia arguta* radix (SAA) and total coumarins of* Peucedani* radix (CPR) were required to suppress the IFN-*γ* and LPS-induced production of NO. The maximal inhibition achieved of SAA and CPR (at 200 mg/L) was 29.69% and 33.65%, respectively (Figure 1).

### 3.2. FGR, FSC, CPR, and SAA-Induced Vasodilation

**Figure 2:**
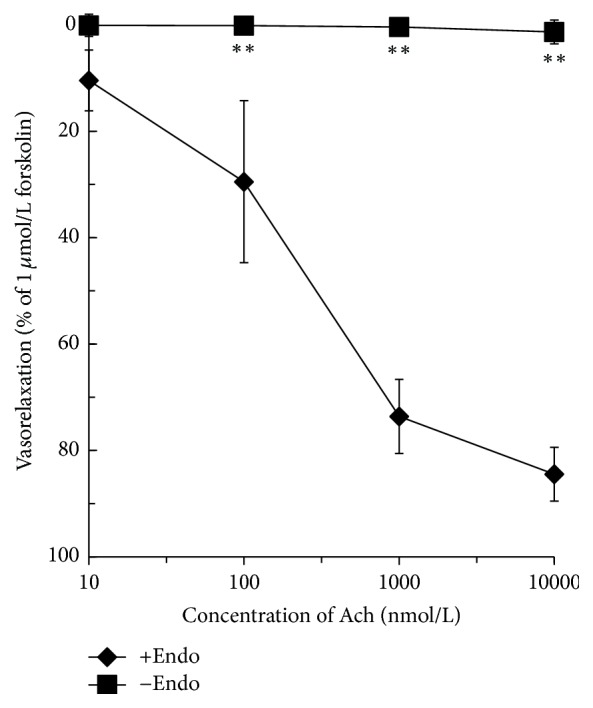
Concentration-response curves showing endothelium-dependent relaxation by Ach with PE pretreated rat aortic rings with intact endothelia (+Endo) and without intact endothelia (−Endo). *n* = 5, ^*∗∗*^*P* < 0.01 versus +Endo.

**Figure 3:**
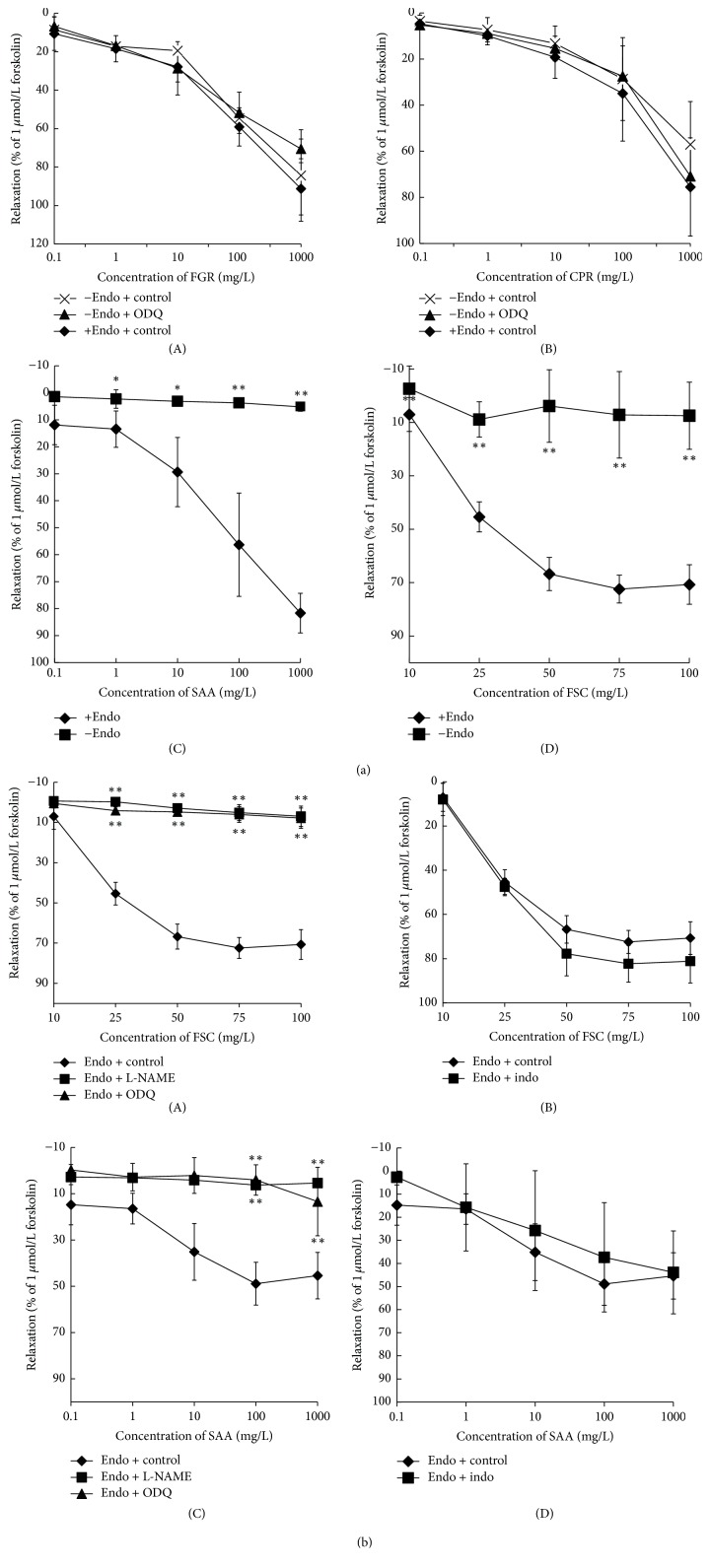
Concentration-response curves showing relaxation by four botanical extracts with PE pretreated rat aortic rings with intact endothelia (+Endo + Control) and without intact endothelia (−Endo + Control). The effects of exposure to 10 *μ*mol/L ODQ on the FSC (b-A), SAA (b-C), FGR (a-A), and CPR (a-B) groups of PE (1 *μ*mol/L) pretreated rings (−Endo + ODQ). The concentration-response curves of FSC (b-B) and SAA (b-D) with pretreatment with Indo. The concentration-response curves of FSC (b-A) and SAA (b-C) with pretreatment with L-NAME. ^*∗*^*P* < 0.05, ^*∗∗*^*P* < 0.01 versus −Endo group, *n* = 4.

Ach-elicited relaxation in aorta rings was used for evaluating intact and deleted endothelium (Figure 2). FGR and CPR relaxed isolated aortic rings in a dose-dependent and endothelium-independent manner. The maximum relaxation by FGR of the aortic rings with or without endothelium was at concentrations of 91.28% ± 5.15% and 84.36% ± 23.80%, respectively. The maximum relaxation by CPR of rings with or without endothelium was at concentrations of 75.51% ± 21.30% and 57.07% ± 18.63%, respectively. The half maximal effective concentration (EC_50_) was 17 mg/L for FGR and 61 mg/L for CPR for aortic rings with absent endothelium as shown in Figure 3(a).

SAA and FSC relaxed isolated aortic rings in a dose-dependent and endothelium-dependent manner. The maximum relaxation of isolated aortic rings by SAA with and without endothelium was at concentrations of 81.66% ± 7.36% and 5.20% ± 1.62%, respectively. The maximum relaxation induced by FSC with and without endothelium was at concentrations of 70.70% ± 6.12% and 7.53% ± 14.08%, respectively. The EC_50_ was 45 mg/L for SAA and 40 mg/L for FSC for aortic rings with intact endothelium as shown in Figure 3(a).

To evaluate the involvement of the NO/cGMP signaling in endothelium-dependent vasodilation, the aortic rings were pretreated with ODQ (10 *μ*mol/L) or L-NAME (100 *μ*mol/L) for 15 minutes each. Soluble guanylate cyclase (sGC) inhibitor ODQ affected FGR and CPR-induced vasodilation (Figure 3(a)). The FSC and SAA-induced relaxations of the aortic tissue were inhibited by pretreatment with ODQ or nitric oxide synthase blocker L-NAME in a concentration-dependent manner (Figure 3(b)).

To investigate the involvement of the cyclooxygenase (COX)/PGI_2_ pathway, one set of aortic tissue was pretreated with indomethacin (10 *μ*mol/L), a nonselective inhibitor of COX. The relaxation curves by FSC or SAA were not significantly altered by the blockage of PGI_2_ pathway (Figure 3(b)).

### 3.3. Effects of FGR and CPR on Endogenous Vasoconstrictors

**Figure 4:**
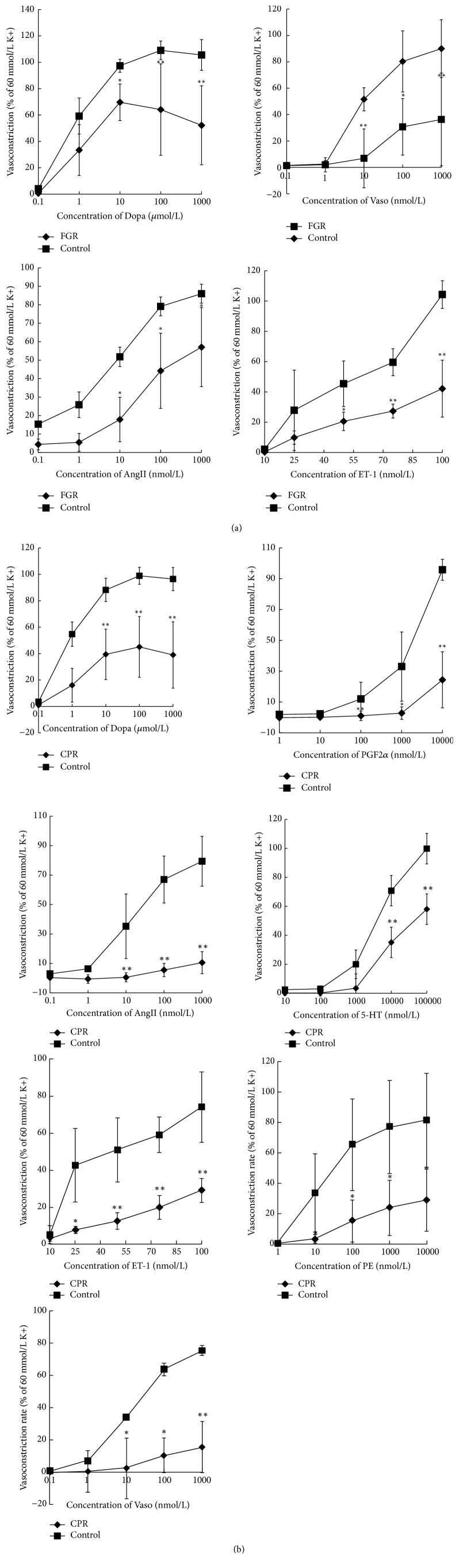
Effects of the four botanical extracts on endothelium-denuded aortic tissue that were exposed to endogenous vasoconstrictors. Inhibited by FGR (a): the contraction curves of Dopa, AngII, Vaso, and ET-1. Inhibited by CPR (b): the contraction curves of Dopa, PGF2*α*, AngII, 5-HT, PE, Vaso, and ET-1. ^*∗*^*P* < 0.05, ^*∗∗*^*P* < 0.01 versus +Endo control group, *n* = 5.

PE, 5-HT, Ang II, ET-1, PG_2*α*_, Vaso, and Dopa are all endogenous vasoconstrictors which play key roles in maintaining vasculature tension [41]. To study endothelium-independent vasodilation, the effects of herb elements on vasocontractility were examined. Aortic rings without endothelium were pretreated with 17 mg/L of FGR and 61 mg/L of CPR, respectively. FGR exerted inhibitory effects on the vasocontraction by Dopa, Ang II, ET-1, and Vaso in a dose-dependent fashion (Figure 4(a)). The maximal inhibitions on vasocontractions by FGR were 38.40%, 50.71%, 59.58%, and 33.67% for Dopa, AngII, ET-1, and Vaso-induced contractilities, respectively. However, FGR failed to suppress vasocontraction induced by PE, PGF, and 5-HT (see Supplemental Figure 1 in Supplementary Material available online at https://doi.org/10.1155/2017/1021284). CPR significantly inhibited vasoconstriction in the presence of Ang II, Dopa, PGF_2*α*_, 5-HT, PE, Vaso, and ET-1 by 86.75%, 59.57%, 74.55%, 41.84%, 64.60%, 79.51%, and 60.55%, respectively (Figure 4(b)).

### 3.4. Effects of FGR and CPR on Potassium Channels

**Figure 5:**
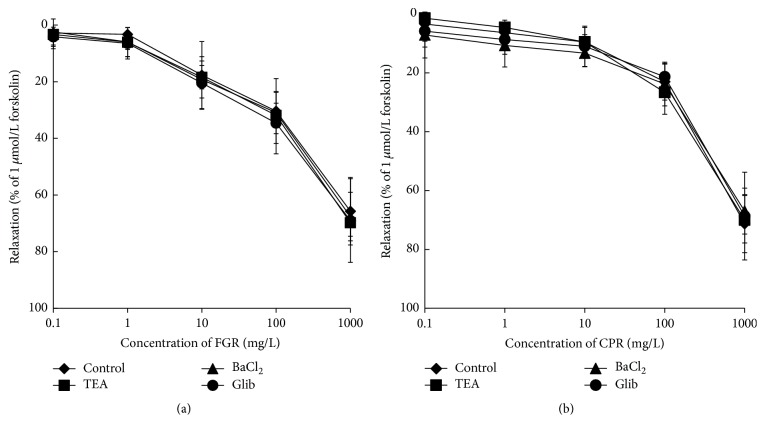
Concentration-response curves showing relaxation induced by FGR (a) and CPR (b) compared to control in endothelium-free tissues pretreated with potassium channel inhibitors: 3 mmol/L TEA, 10 *μ*mol/L Glib, and 100 *μ*mol/L BaCl_2_, *n* = 6.

Potassium channels are important to vascular relaxation. There are many types of potassium channels in vascular smooth muscle including calcium-activated potassium channel (K_Ca_), ATP-sensitive K+ channels (K_ATP_), and inwardly rectifying potassium channels (K_IR_). To test the possible involvement of K^+^ channels in relaxations induced by FGR and CPR, endothelium-denuded rings were preincubated with K_Ca_ blocker (TEA) at 100 mmol/L, K_ATP_ blocker (glibenclamide) at 10 mmol/L, and K_IR_ blocker BaCl_2_ at 100 mmol/L, respectively, for 15 minutes. In each case, the FGR- and CPR-induced vascular relaxation was not inhibited by glibenclamide, TEA, or BaCl_2_. Glibenclamide, TEA, or BaCl2 did not inhibit vascular relaxation by FGR. We also used glibenclamide, TEA, or BaCl_2_ to preincubate the endothelium-denuded rings, which did not inhibit vascular relaxation induced by CPR (Figure 5).

### 3.5. Effects of FGR and CPR on Extracellular Calcium Influx and Intracellular Calcium Release

**Figure 6:**
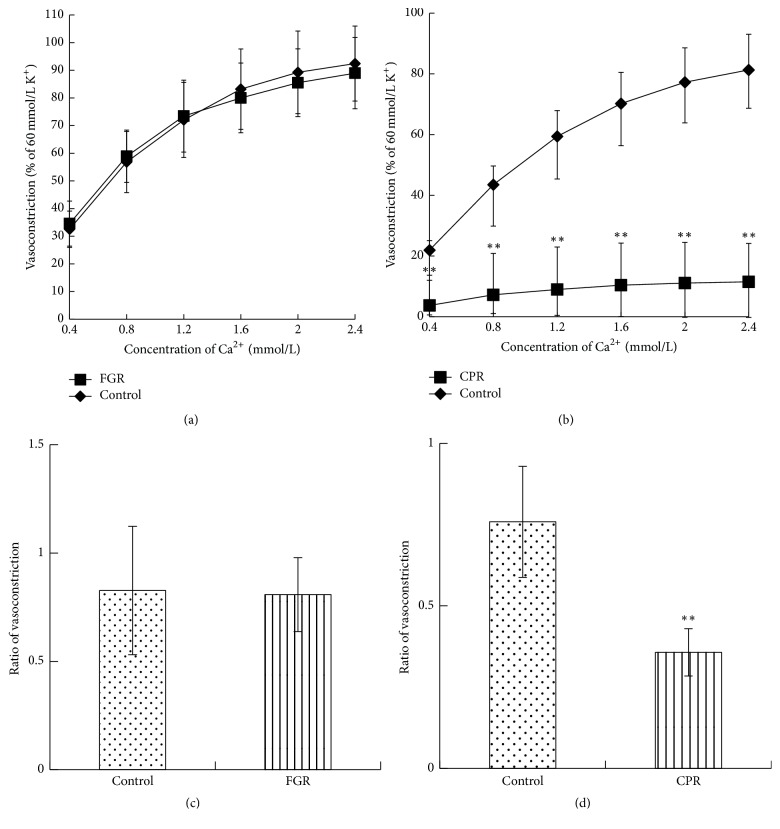
Effects of four botanical extracts on calcium channel and cytoplasmic calcium release. The concentration-response curves of CaCl_2_ in Ca^2+^-free media were inhibited by FGR (a) and CPR (b); maximal (100%) contraction was represented by 60 mmol/L KCl-induced contractions. Effects of four botanical extracts on the transient contraction induced by PE in Ca^2+^ free media. The effect of PE in Ca^2+^ free media was inhibited by FGR (c) and CPR (d); maximal (100%) contraction was represented by 60 mmol/L KCl-induced contraction. ^*∗∗*^*P* < 0.01 versus control, *n* = 5–7.

Endogenous vasoconstrictors, such as PE, contract vascular smooth muscle mainly through the activation of receptor-operated calcium channels (ROCC), while KC1 mainly activates potential-dependent Ca2+ channels, all of which result in both extracellular calcium influx and intracellular calcium release. To confirm whether calcium-mediated vasoconstriction is affected by FGR and CPR, aortic ring samples denuded of endothelium were exposed to Ca^2+^-free K-H solutions, and the addition of 1 *μ*mol/L PE induced small tonic contractions which were most likely activated by the release of intracellular Ca^2+^ from endoplasmic reticulum stores. CPR reduced PE-induced contractions better than FGR under extracellular Ca^2+^-free condition (Figure 6).

Experiments on depolarization elicited by voltage-dependent Ca^2+^-influx in high concentrations of K^+^ were tested as shown in Figure 6. The data suggested that the K^+^ (60 mmol/L) stimulated, Ca^2+^-induced vasoconstriction was not inhibited by 17 mg/L of FGR. However, the vasoconstriction was suppressed by 61 mg/L of CPR.

## 4. Discussion

The total saponins from* Actinidia arguta* radix (SAA), total flavonoids from* Glycyrrhizae *radix et rhizoma (FGR), total coumarins from* Peucedani* radix (CPR), and total flavonoids from* Spatholobi* caulis (FSC) were extracted and used in current studies. Four anti-inflammatory herbal extracts relaxed thoracic aortic ring in a concentration-dependent manner. The rank order of the EC_50_ for relaxation of these extracts was as follows:* Glycyrrhizae* radix et rhizoma <* Spatholobi* caulis <* Actinidia arguta* radix <* Peucedani* radix.

The vascular relaxation evoked by SAA is endothelium dependent and the vasodilatory effect by the element from Radix and Stemma Actinidia argute (Teng Li Gen) is clocked by ODQ, a soluble guanylyl cyclase (sGC) inhibitor. Thus, our study first revealed that a NO-cGMP dependent pathway is critical for the action of the SAA. As a major component of saponin from the Actinidia argute, it has been known that corosolic acid possesses various biological properties, including antidiabetic, antiobesity, and anti-inflammatory activities [42–44] The compound's efficacy in diabetes has resulted the development of Glucosol (or GlucoFit), a commercially available product primarily marketed in Japan and the United States as a dietary supplement for weight loss and blood sugar balance. The inflammatory and oxidative stress impact metabolism through lipid and glucose metabolism and insulin resistance which is linked to mitochondrial function [10]. TEO (2a,-3a,-24-trihydroxyurs-12-en-28-oic acid), a corosolic acid analogue, declined the mitochondrial membrane potential and altered mitochondrial ultrastructure which may serve the mechanism for the antioxidative stress effects [45]. Nevertheless, cGMP has been reported to exert an action on mitochondrial function [46]. On the other hand, corosolic acid has been shown to suppress glioblastoma cell proliferation by inhibiting the activation of signal transducer and activator of transcription-3 and nuclear factor-kappa B in tumor cells and tumor-associated macrophages corosolic acid inhibits glioblastoma cell proliferation [47]. Our analysis of GEO databases (National Cancer Institute) revealed a statistically significant reduction of sGC transcript levels in human glioma specimens. Pharmacologically manipulating endogenous cGMP generation in glioma cells through either stimulating pGC by ANP/BNP or blocking PDE by 3-isobutyl-1-methylxanthine/zaprinast caused significant inhibition of proliferation and colony formation of glioma cells. Our study proposes the new concept that suppressed expression of sGC, a key enzyme in the NO/cGMP pathway, may be associated with an aggressive course of glioma. The sGC/cGMP signaling-targeted therapy may be a favorable alternative to chemotherapy and radiotherapy for glioma and perhaps other tumors [48].

The relaxation induced by FSC was inhibited by L-NAME, indicating the involvement of NO in vascular dilatory action of the extracts.* Spatholobi* caulis is a traditional blood-activating and stasis-dispelling herb medicine, which has been used to treat diseases related to blood stasis syndrome by inhibiting platelet aggregation and stimulating hematopoiesis. A recent study further revealed that the FSC presented proangiogenic activity in human umbilical vein endothelial cells (HUVECs) as well as in zebrafish [49]. With an LPS-activated Raw264.7 cells model, the* Spatholobi* caulis MeOH extract (containing flavonoids) inhibited the expressions of iNOS and COX-2 and suppressed the production of proinflammatory cytokines, such as IL-1beta and IL-6 [50]. Genistein, an isoflavonoid from the herb, has been reported to decrease the generations of ROS and malondialdehyde [51]. In mammalian cells, NO is produced by a family of NO synthases (NOS). Three NOS isoforms have been identified as neuronal NOS (nNOS), inducible NOS (iNOS), and endothelial NOS (eNOS). In vascular system, NO is generated from the conversion of L-arginine to L-citrulline by eNOS, which requires Ca^2+^/calmodulin, FAD, FMN, and tetrahydrobiopterin (BH_4_) as cofactors. Under the inflammatory pathological conditions, the cofactors of eNOS can be oxidized and eNOS then shifts to produce superoxide anion instead of NO. This state is referred to as the “uncoupled state of eNOS” (eNOS uncoupling), which may further enhance the inflammation [52]. Considering the significant anti-inflammatory effect of the FSC which markedly inhibited the expressions of iNOS and proinflammatory cytokines, we speculate that the vasodilatory effect of the FSC may be partially due to its promoting of eNOS function through antioxidative properties.

Radix* Glycyrrhizae *(Licorice Root) is the most used herb element in TCM. Licorice, the root extract of Glycyrrhiza glabra I., is used as a medicine for various diseases. Anti-inflammatory as well as antiallergic activities have been attributed to one of its main constituents, glycyrrhizin. These activities are mainly ascribed to the action of the aglycone, beta-glycyrrhetinic acid. beta-Glycyrrhetinic acid has a steroid-like structure and is believed to have immunomodulatory properties [53]. Glycyrrhizin inhibits liver cell injury and is given intravenously for the treatment of chronic viral hepatitis and cirrhosis in Japan [54, 55]. It has also proven itself effective in the treatment of autoimmune hepatitis in one clinical trial [56]. We demonstrate a significant vasodilatory effect of FGR (total flavonoids from Glycyrrhizae radix et rhizoma) and reveal that pretreatment with FGR shifted contraction curves of Dopa, AngII, Vaso, and ET-1 to the right. Those endogenous vasoconstrictors regulate vascular tone via their respective receptors (mostly G protein-coupled) in smooth muscle. Although overall mechanisms of action are different, G protein-coupled receptors as a whole activate PLC, DAG, and IP3. DAG elicits protein kinase C by activating myosin light chains. IP3 induces intracellular calcium release from the intracellular calcium pool or activates VDCCs in the cell membrane to regulate intracellular calcium concentration and vascular tone [57]. However, FGR failed to block Ca^2+^ influx or releasing intracellular Ca^2+^. Glycyrrhetic acid, the active metabolite in licorice, inhibits the enzyme 11-*β*-hydroxysteroid dehydrogenase enzyme type 2 with a resultant cortisol-induced mineralocorticoid effect and the tendency towards the elevation of sodium and reduction of potassium levels. This aldosterone-like action is the fundamental basis for understanding the pharmacology of the extract [58]. However, the glucocorticoids inhibits eNOS gene expression and reduces NO release through the glucocorticoid receptor mediated signaling [59]. The glucocorticoids also directly potentiate contractions of rabbit and dog aortic strips to epinephrine and norepinephrine [60, 61]. Thus, the specific mechanisms underlying relaxation of vascular smooth muscle by FGR need further study.

Khellactone (dihydroseselin) coumarins possess various activities, including calcium blocker and antiplatelet aggregation [62, 63]. Khellactone coumarins with 3′S, 4′S configuration (praeruptorins A, B, C, and D) were first isolated from dried roots of P. praeruptorum (*Peucedani* radix) which is commonly used in Traditional Chinese Medicine (TCM) for treatment of cough and upper respiratory infections and as an antipyretic, antitussive, and mucolytic agent. By using spontaneously hypertensive rats as experimental model, praeruptorin-C improved the vascular hypertrophy by decreasing the size of SMCs cells, collagen content, and increasing NO production [64]. The vasodilatory effects of praeruptorin-A was confirmed in isolated rabbit tracheas and pulmonary arteries, as well as in swine coronary artery [65, 66]. In our experimental setting, total coumarins from* Peucedani* radix (CPR) induced vascular relaxation may not be related to sGC/cGMP but is associated with blocking of both VDCC and ROCC.

## 5. Conclusion

The present study shows that extracts from four herbs relaxed thoracic aorta tissues isolated from rats.* Glycyrrhizae *radix et rhizome and* Peucedani *radix induced vasorelaxation independent of intact endothelium; however, their respective mechanisms of action appear to be different. Vasorelaxation induced by* Peucedani *radix appears to be mainly related to effects on intracellular calcium homeostasis, specifically the inhibition of Ca^2+^ influx and intracellular Ca^2+^ release. Dopa-, AngII-, Vaso-, and ET-1 induced vasoconstriction was inhibited by* Glycyrrhizae* radix et rhizome, but details of its mechanism of action need further study. The vasorelaxation induced by* Spatholobi *caulis and* Actinidia arguta* radix is endothelium-dependent, and their mechanisms of relaxation may involve the NO-cGMP pathway. The distinct vasodilatory effects of four anti-inflammatory botanical extracts are significant and novel which will pave the way not only for further mechanism study, but also for directing of new herb formula for preventive and/or therapeutic usage.

## Supplementary Material

Supplemental Figure 1. FGR failed to suppress vasocontraction induced by PE, PGF, and 5-HT.

## Figures and Tables

**Table 1:** Botanical extracts that possess anti-inflammatory properties.

Medicinals	Photos	Nature of medicinals	Flavor of medicinals	Functions	Clinical application	Reference
*Spatholobus suberectus *Dunn	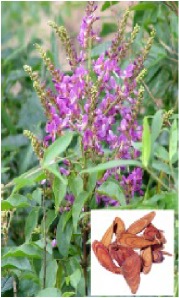	Warm	Bitter, sweet	Promoting blood flow, tonifying blood, regulating menstruation and relieving pain, relaxing sinews, and activating collaterals	Treatment of irregular menstruation, dysmenorrhoea, amenorrhea, rheumatic arthralgia, paralysis or numbness in the limbs, blood deficiency, and chlorosis	Chinese Pharmacopoeia (Version 2015)

*Peucedanum praeruptorum *Dunn	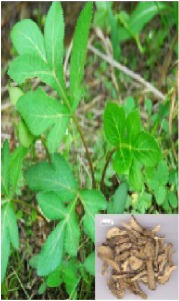	Slightly cold	Bitter, pungent	Directing qi downward to resolve phlegm, dispelling pathogenic wind, and clearing heat	Treatment of respiratory symptoms such as phlegm-heat asthma, yellow viscous phlegm, cough, and phlegm induced by wind-heat	Chinese Pharmacopoeia (Version 2015)

*Actinidia arguta *(Sieb. &Zucc.) Planch. ex Miq	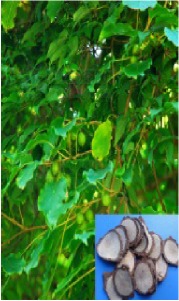	Cool	Sour, astringent	Clearing heat and detoxicating, dispelling wind-dampness, diuretic, and haemostatic	Treatment of rheumatism, pain, and jaundice	The national compilation of Chinese herbal medicine

*Glycyrrhiza uralensis *Fisch	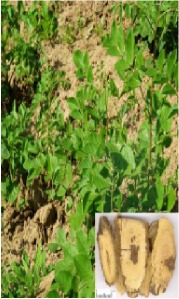	Calm	Sweet	Tonifying spleen and qi, clearing heat and detoxicating, dispelling phlegm and suppressing cough, relieving spasm and pain, mixed herbs	Treatment of deficiency of spleen and stomach, fatigue, palpitations, shortness of breath, cough and phlegm in throat, abdominal pain, spasm of limbs, carbuncle sore, and reduce drug toxicity	Chinese Pharmacopoeia (Version 2015)
