# Supplementary material for: The Vasodilatory Effects of Anti-Inflammatory Herb Medications: A Comparison Study of Four Botanical Extracts
Source: Evid Based Complement Alternat Med. 2017 Nov 28;2017:1021284. doi: 10.1155/2017/1021284 (PMC5733232; doi:10.1155/2017/1021284)
Supplement: Supplementary file 1 — Supplemental Figure 1. FGR failed to suppress vasocontraction induced by PE, PGF, and 5-HT. [file 1021284.f1.pdf]

Supplemental Fig.1

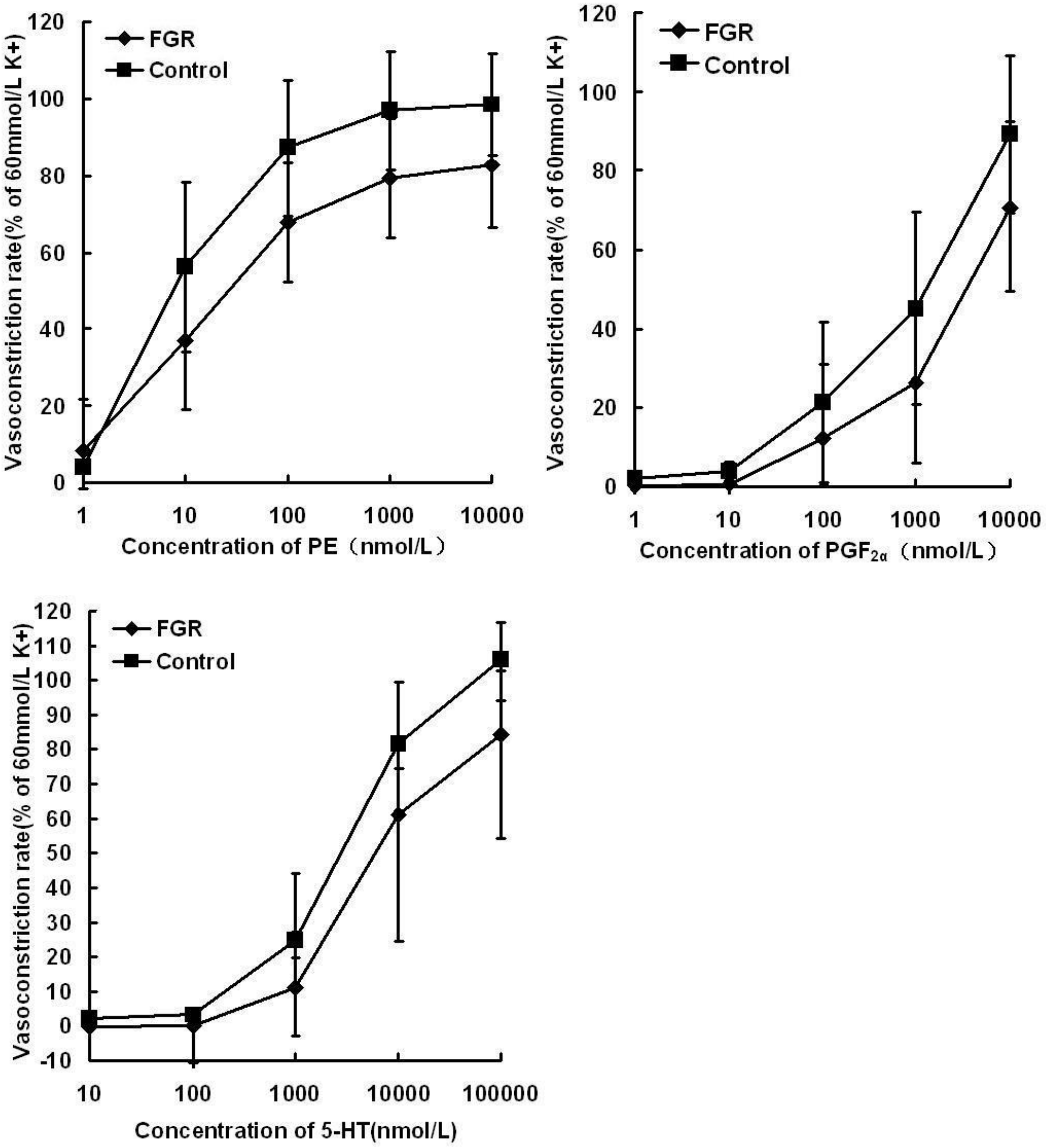

Supplemental Fig.1 Effects of FGR on endothelium-denuded aortic tissue that were exposed to PE, $PGF_{2\alpha}$, and 5-HT, n =5.
